# The Expression of Cold-Inducible RNA-Binding Protein mRNA in Sow Genital Tract is Modulated by Natural Mating, but not by Seminal Plasma

**DOI:** 10.3390/ijms21155333

**Published:** 2020-07-27

**Authors:** Jaume Gardela, Mateo Ruiz-Conca, Cristina A. Martinez, Dominic Wright, Manel López-Béjar, Heriberto Rodriguez-Martinez, Manuel Alvarez-Rodriguez

**Affiliations:** 1 Department of Biomedical and Clinical Sciences (BKV), Division of Children’s and Women Health (BKH), Obstetrics and Gynaecology, Linköping University, 58185 Linköping, Sweden; jaume.gardela@uab.cat (J.G.); mateo.ruiz@uab.cat (M.R.-C.); cristina.martinez-serrano@liu.se (C.A.M.); heriberto.rodriguez-martinez@liu.se (H.R.-M.); 2 Department of Animal Health and Anatomy, Veterinary Faculty, Universitat Autònoma de Barcelona, 08193 Bellaterra, Spain; manel.lopez.bejar@uab.cat or; 3 Department of Physics, Chemistry and Biology, Faculty of Science and Engineering, Linköping University, 58185 Linköping, Sweden; dominic.wright@liu.se; 4 College of Veterinary Medicine, Western University of Health Sciences, Pomona, CA 91766, USA

**Keywords:** transcriptomics, microarrays, spermatozoa, seminal plasma, cold-inducible proteins, cold-sensitive TRP ion channels, pig

## Abstract

The RNA-binding proteins (RBPs), some of them induced by transient receptor potential (TRP) ion channels, are crucial regulators of RNA function that can contribute to reproductive pathogenesis, including inflammation and immune dysfunction. This study aimed to reveal the influence of spermatozoa, seminal plasma, or natural mating on mRNA expression of RBPs and TRP ion channels in different segments of the internal genital tract of oestrous, preovulatory sows. Particularly, we focused on mRNA expression changes of the cold-inducible proteins (CIPs) and related TRP channels. Pre-ovulatory sows were naturally mated (NM) or cervically infused with semen (Semen-AI) or sperm-free seminal plasma either from the entire ejaculate (SP-TOTAL) or the sperm-rich fraction (SP-AI). Samples (cervix to infundibulum) were collected by laparotomy under general anaesthesia for transcriptomic analysis (GeneChip^®^ Porcine Gene 1.0 ST Array) 24 h after treatments. The NM treatment induced most of the mRNA expression changes, compared to Semen-AI, SP-AI, and SP-TOTAL treatments including unique significative changes in *CIRBP*, *RBM11*, *RBM15B*, *RBMS1*, *TRPC1*, *TRPC4*, *TRPC7*, and *TRPM8*. The findings on the differential mRNA expression on RBPs and TRP ion channels, especially to CIPs and related TRP ion channels, suggest that spermatozoa and seminal plasma differentially modulated both protein families during the preovulatory phase, probably related to a still unknown early signalling mechanism in the sow reproductive tract.

## 1. Introduction

Mammals reproduce by internal fertilization releasing billions of spermatozoa into the female reproductive tract to achieve maximum chances of fertilization, despite only thousands reaching the oviduct [1], where fertilization takes place. Ejaculate deposition into the female reproductive tract affects the molecular and cellular function of reproductive organs proximal and distal to the insemination site [2]. The interaction between the female reproductive tract and semen induces mRNA and protein expression changes in endometrial and oviductal tissues modulating important processes for reproduction, such as angiogenesis, sperm storage and selection, oviduct contractility and oocyte transportation, and preimplantation embryo development, previously reported in pigs [3,4,5,6] and other species [7,8,9,10,11].

Many RNA-binding proteins (RBPs) are involved in the control of inflammation and immune dysfunction, which can contribute to pathogenesis of reproductive disorders [12]. Each step of RNA metabolism (transcription, splicing, polyadenylation, stabilization, edition, capping, or translation) is assisted by the RBPs [13]. These proteins are the most important regulators of RNAs and crucial for gene regulation, containing different structural RNA-binding motifs or domains, such as RNA recognition motif (RRM), K homology domain, double-stranded RNA binding domain, or zinc fingers, forming ribonucleoprotein complexes by binding double or single-stranded RNA [14]. Beyond their RNA-binding domains, the RBPs hold many structural modules that facilitate protein-protein interactions and catalytic events; thereby, they are incorporated in numerous intracellular processes [14].

The cold-inducible proteins (CIPs) are evolutionarily conserved RBPs that have been detected in multiple organisms from different taxa [15]. The cold-inducible RNA-binding protein (CIRBP, also known as CIRP, or heterogenous nuclear ribonucleoprotein A18, hnRNP A18), the RNA-binding motif protein 3 (RBM3), and the serine and arginine-rich splicing factor 5 (SRSF5, also known as SRp40) are CIPs transcriptionally up-regulated in response to moderately low temperatures and other cellular stressors, such as hypoxia, DNA damage, or osmotic stress [16,17].

CIRBP was first described in the late 1990s as a novel RBP which expression was induced in mouse cells in response to cold stress [18]. Since this discovery, investigations revealed its role in many cellular stress responses, such as mRNA stability, cell survival, proliferation, stress adaptation, and tumor formation [19]. Intracellularly, CIRBP acts as a RNA chaperone to facilitate translation, whereas extracellularly, it seems to play a crucial role in the promotion of inflammation and injury [20,21].

Both CIRBP and RBM3 are structurally similar to the heterogeneous nuclear ribonucleoproteins and belong to the glycine-rich RBP family class IVa [15], which contains, at the *N*-terminal end, one conserved RRM and two ribonucleoprotein domains (RNPs), known as RNP1 and RNP2 [17]. The C-terminal contains a less conserved arginine-glycine-rich (RGG) domain, and this is the reason why CIRBP and RBM3 belong to the large family of glycine-rich proteins [17]. SRSF5 belongs to the serine/arginine-rich family protein (also called serine/arginine-rich splicing factors, SRSFs), characterized by the presence of one or two *N*-terminal RRMs followed by a downstream domain rich in arginine and serine residues [22]. Heterogeneous nuclear ribonucleoproteins and SRSFs are both active factors involved in the regulation of pre-mRNA splicing, and due to its derived origin from a common ancestor, have been suggested to play an important role in the evolution of the alternative splicing mechanism [23]. Through alternative splicing, a single gene can encode for multiple variants, greatly enhancing the transcriptome complexity, the variety of proteins and hence, the diverse biological functions [24].

The induction of CIPs is dependent on transient receptor potential (TRP) V4, V3, and M8 ion channel proteins [16,25]. The TRP mammalian proteins can be classified in four major families [26]: the ankyrin-related TRP channel family (TRPA), the TRPC family (classical or canonical), the melastatin-related TRP channel family (TRPM), and the channels homologous to vanilloid receptor (TRPV). These receptors are non-selective cation channels permeable for Ca^2+^ that open in response to multiple chemical and physical stimuli [27,28,29], providing Ca^2+^ influx pathways [26]. Most of them have thermosensitive abilities, like TRPM8, TRPA1, and TRPC5, which have been proposed as cold-sensitive ion channels [29].

Even though current research has focused on discerning the effects produced by spermatozoa and seminal plasma (SP) in endometrium and oviductal mRNA expression in the sow reproductive tract [30,31,32], to the best of our knowledge, there is a lack of literature regarding the differential mRNA expression levels of genes encoding for RBPs and TRP ion channels. Here, we specifically studied the differential mRNA expression levels of CIPs and related TRP ion channels in the internal genital tract of pre-ovulatory sows induced by the interaction of semen (spermatozoa and/or SP) with the genital mucosa.

## 2. Results

The presence of inflammation was analyzed by the mRNA expression of the interleukin-8, also called CXCL8, a chemokine induced in lipopolysaccharide-stimulated monocytes inducing neutrophil migration [33] and often used as classical inflammation marker. In this study, mRNA expression of *CXCL8* was down-regulated (*p* < 0.05) by natural mating (NM) in the cervix (fold change −4.38) and the proximal uterus (fold change −3.06), and down-regulated (*p* < 0.05) by artificial insemination with the sperm-peak fraction (Semen-AI) in the utero-tubal junction (fold change −5.78), whereas no differences were found in the rest of the tissues and treatments. Spermatozoa were only present in the utero-tubal junction of NM and Semen-AI treated animals but not in animals treated with sperm-free SP or controls.

### 2.1. Induced mRNA Expression Changes on RBPs and TRP Ion Channels

Table 1 displays the mRNA expression levels of the 39 differentially expressed genes (DEGs) encoding for RBPs and TRP ion channels for each treatment and tissue sample.

**Figure 1:**
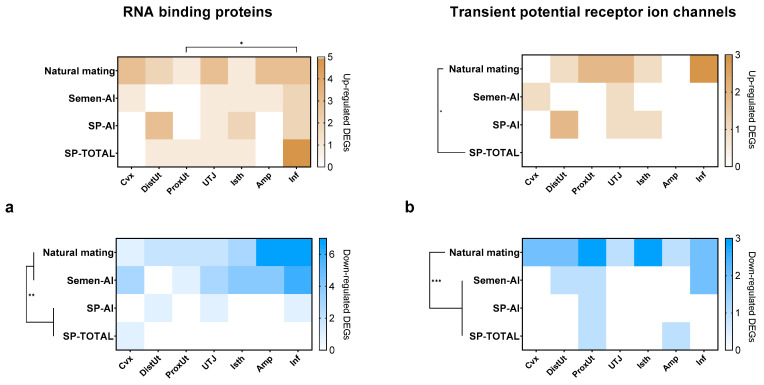
Heatmap composition of the number of differentially expressed genes (*p* < 0.05) in the porcine female reproductive tract 24 h post-treatment. Genes belonging to: (**a**) the RNA-binding protein family that contain the RNA recognition motif domain (IPR000504) in their structure, and (**b**) the transient receptor potential ion channels, up-regulated (brown) and down-regulated (blue). Cvx: cervix; DistUt: distal uterus; ProxUt: proximal uterus; UTJ: utero-tubal junction, Isth: isthmus; Amp: ampulla; Inf: infundibulum. Natural mating: sows mated with a boar; Semen-AI: sows artificially inseminated with the sperm-peak portion extended to 50 mL with Beltsville thawing solution (BTS); SP-AI: sows cervically infused with the sperm-free seminal plasma (SP) from pooled sperm-peak portion (50 mL); SP-TOTAL: sows cervically infused with the sperm-free SP of the whole ejaculate (50 mL). All treatments were compared with controls (cervical infusion with 50 mL BTS). Asterisks represent statistical differences between treatments and tissues (* *p* < 0.05, ** *p* < 0.01, *** *p* < 0.001).

The number of DEGs (up- and down-regulated, *p* < 0.05), with respect to the control group (cervical infusion with 50 mL Beltsville thawing solution, BTS), that codify for RBP family containing the RRM domain (IPR000504) were displayed in Figure 1a. Even though the statistical analyses did not determine overall differences among groups in the number of up-regulated DEGs encoding for RBPs (NM: 16 genes, Semen-AI: 6 genes, sperm-free SP infusion from the sperm-peak fraction (SP-AI): 8 genes, and sperm-free SP infusion from the whole ejaculate (SP-TOTAL): 9 genes; *p* > 0.05), the infundibulum showed a greater number of up-regulated DEGs encoding for RBPs (12 genes, *p* < 0.05) compared to the proximal uterus (2 genes), within all treatments. Sperm-containing treatments down-regulated a greater number of DEGs encoding for RBPs (NM: 24 genes, and Semen-AI: 21 genes; *p* < 0.01) compared to sperm-free SP treatments (SP-AI: 3 genes, and SP-TOTAL: 1 gene).

Figure 1b displays the number of DEGs (up- and down-regulated, *p* < 0.05) that codify for TRP ion channels. A greater number of DEGs encoding for TRP ion channels were up-regulated by NM (9 genes, *p* < 0.05) compared to SP-TOTAL (0 genes). The down-regulated DEGs encoding for TRP ion channels were significantly affected by NM (14 genes, *p* < 0.001) compared to the rest of the treatments (Semen-AI: 4 genes, SP-AI: 1 gene, and SP-TOTAL: 2 genes).

**Figure 2:**
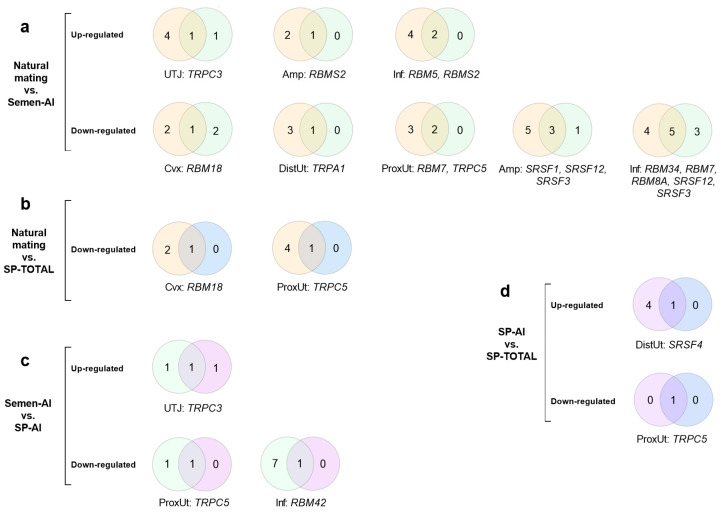
Venn diagrams of commonly differentially expressed genes (*p* < 0.05) in the porcine female reproductive tract codifying for RNA-binding proteins and transient receptor potential ion channels (up- or down-regulated), 24 h post-treatment. (**a**) Sows mated with a boar (Natural mating, orange) or sows artificially inseminated with the sperm-peak portion extended to 50 mL with Beltsville thawing solution (BTS) (Semen-AI, green); (**b**) Natural mating (orange) or sows cervically infused with the sperm-free SP of the whole ejaculate (50 mL) (SP-TOTAL, blue); (**c**) Semen-AI (green) sows cervically infused with the sperm-free seminal plasma (SP) from pooled sperm-peak portion (50 mL) (SP-AI, purple); (**d**) SP-AI (purple) or SP-TOTAL (blue). Cvx: cervix, ProxUt: proximal uterus, DistUt: distal uterus, UTJ: utero-tubal junction, Amp: ampulla, and Inf: infundibulum. The acronyms of genes common to treatment per tissue (crossing sectors) are identified.

The number of common DEGs that codify for RBPs and TRP ion channels (up- and down-regulated, *p* < 0.05) in the reproductive tract of sows between treatments were displayed in a series of Venn diagrams [34], indicating which DEGs were identified as common to each treatment per tissue (Figure 2). As may be seen, the mRNA expression pattern was affected by the entrance of the early sperm-rich fraction of the ejaculate in the female reproductive tract (Figure 2a). The common DEGs between NM and Semen-AI groups tended to be down-regulated (12 down-regulated DEGs vs. 4 up-regulated DEGs, Figure 2a). The combination between NM and SP-TOTAL groups only demonstrated common down-regulated DEGs in the uterus (Figure 2b). Combinations including sperm-free SP treatments showed common up- and down-regulated DEGs generally observed in the uterus (Figure 2b–d). However, the combination between Semen-AI and SP-AI groups showed common up- and down-regulated DEGs in the oviduct (Figure 2c). The combination between NM and Semen-AI groups showed a predominant number of up- and down-regulated DEGs in the oviductal segments compared to the uterine segments (11 DEGs in oviductal tissues vs. 5 DEGs in uterine segments, Figure 2a). Curiously, the proximal uterus showed a common down-regulation of *TRPC5* expression in all combinations. Additionally, the utero-tubal junction showed a common up-regulation of *TRPC3* between NM and Semen-AI groups, and SP-AI and Semen-AI groups (Figure 2a,c).

### 2.2. mRNA Expression Changes on CIPs and Related TRP Ion Channels

Of the total number of DEGs encoding for RBP family included in the study (26 genes, Table 1), only two genes belong to the CIPs: *CIRBP* and *SRSF5* [16,17]. The NM group up-regulated *CIRBP* mRNA expression in the cervix, utero-tubal junction, ampulla, and infundibulum (*p* < 0.05), and up-regulated *SRSF5* mRNA expression in the utero-tubal junction (*p* < 0.05), whereas Semen-AI down-regulated *SRSF5* mRNA expression in the cervix (*p* < 0.05) and SP-AI up-regulated *SRSF5* mRNA expression in the infundibulum (*p* < 0.05).

Of the total number of DEGs encoding for TRP ion channels included in the study (13 genes, Table 1), only three genes encode for the cold-sensitive TRP ion channels: *TRPA1, TRPC5*, and *TRPM8* [29]. The NM group down-regulated *TRPC5* mRNA expression in all tissues (*p* < 0.05). As previously stated, all treatments down-regulated *TRPC5* mRNA expression in the proximal uterus (*p* < 0.05). Besides, NM down-regulated *TRPA1* mRNA expression in the uterus and isthmus (*p* < 0.05), and down-regulated *TRPM8* mRNA expression in the isthmus (*p* < 0.05). Additionally, Semen-AI down-regulated *TRPA1* mRNA expression in the distal uterus (*p* < 0.05).

Three genes encoding for TRP ion channels are involved in the induction of CIPs: *TRPM8*, *TRPV3*, and *TRPV4* [16,25]. No statistical differences were found for *TRPV3* and *TRPV4* mRNA expression.

### 2.3. Gene Ontology and Kyoto Encyclopedia of Genes and Genomes Analyses

**Figure 3:**
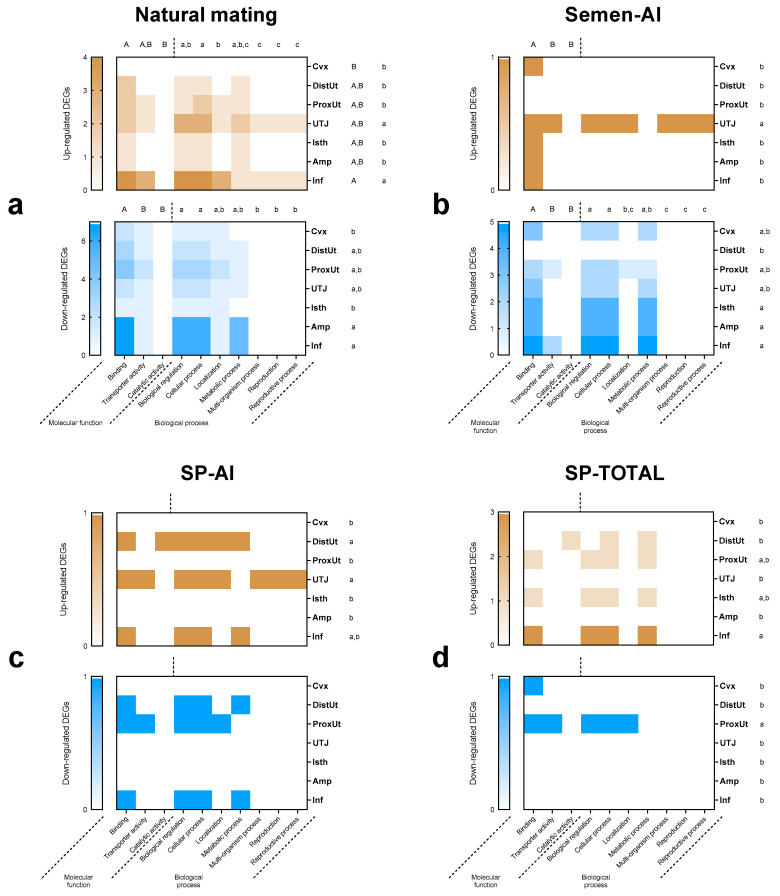
Heatmap composition of Gene Ontology (GO) classification. Categories of the differentially expressed genes (*p* < 0.05), up-regulated (brown) and down-regulated (blue), based on GO function along the segments of the internal female genital tract (Cvx: cervix; DistUt: distal uterus; ProxUt: proximal uterus; UTJ: utero-tubal junction, Isth: isthmus; Amp: ampulla; Inf: infundibulum) after the different treatments: (**a**) Natural mating (sows mated with a boar), (**b**) Semen-AI (sows artificially inseminated with the sperm-peak portion extended to 50 mL with Beltsville thawing solution (BTS)), (**c**) SP-AI (sows cervically infused with the sperm-free SP from pooled sperm-peak portion (50 mL)), and (**d**) SP-TOTAL (sows cervically infused with the sperm-free seminal plasma (SP) of the whole ejaculate (50 mL)). The results are summarized in two major GO categories: molecular function and biological process. Uppercase letters represent differences between molecular function GO categories (*p* < 0.05) and lowercase letters represent differences between biological process GO categories (*p* < 0.05). Differences between tissues in molecular function GO categories are represented in uppercase letters (*p* < 0.05) and differences between tissues in biological process GO categories are represented with lowercase letters (*p* < 0.05).

To categorize the function of the 39 DEGs detected, genes were mapped to terms in Gene Ontology (GO) database [35] (Figure 3). Out of the two main categories of the GO classification, the “biological process” was dominant. The predominant molecular function in the sperm-containing treatments was binding activity (GO:0005488) in both up- and down-regulated genes (*p* < 0.01). The predominant biological processes of the down-regulated genes in the sperm-containing treatments were biological regulation (GO:0065007) and cellular process (GO:0009987) followed by metabolic process (GO:0008152) (*p* < 0.01). The predominant biological process in the genes up-regulated by NM was cellular process (GO:0009987), followed by biological regulation (GO:0065007) (*p* < 0.05). No statistical differences were found within molecular functions and biological processes in the sperm-free SP treatments (*p* > 0.05). Molecular functions only differed in tissues where genes were up-regulated by the NM treatment (Figure 3a) predominantly in the infundibulum compared to endocervix (*p* < 0.05). The biological processes of the genes up-regulated by NM (Figure 3a) were mostly in the infundibulum and utero-tubal junction compared to the rest of the tissues (*p* < 0.05), whereas the biological processes of the genes down-regulated by NM (Figure 3a) were dominant in the ampulla and infundibulum compared to the endocervix and isthmus (*p* < 0.05). Similarly, the biological processes of the genes up-regulated by Semen-AI (Figure 3b) were dominant in the utero-tubal junction compared to the rest of the tissues (*p* < 0.001), whereas the biological processes of the genes down-regulated by Semen-AI (Figure 3b) dominated in the isthmus, ampulla, and infundibulum compared to the distal uterus (*p* < 0.05). The biological processes of the SP-AI group (Figure 3c) only differed among up-regulated genes, these being dominant in the distal uterus and utero-tubal junction compared to the endocervix, proximal uterus, isthmus, and ampulla (*p* < 0.05). Conversely, the biological processes of the genes up-regulated by SP-TOTAL (Figure 3d) appeared mostly in the infundibulum compared to the endocervix, distal uterus, utero-tubal junction, and ampulla (*p* < 0.05), whereas the genes down-regulated by SP-TOTAL (Figure 3d) dominated the proximal uterus compared to the rest of the tissues (*p* < 0.01).

**Figure 4:**
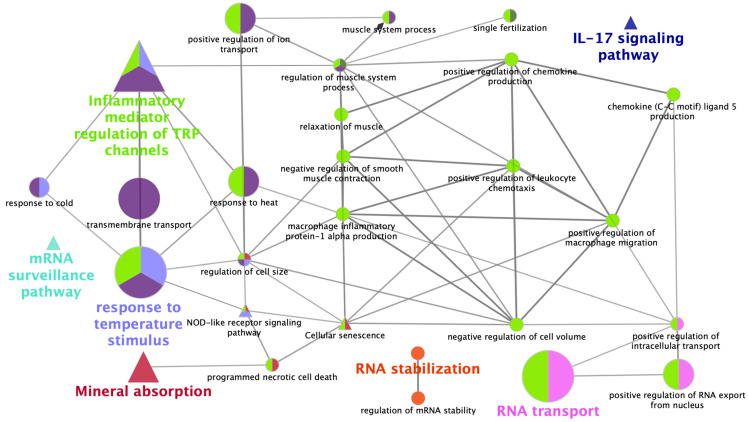
Schematic representation of selected altered transcripts among all tissues and treatments. The analysis of over-represented functional categories was performed using the Cytoscape v3.0.0 application ClueGo v2.0.3. The following databases were used: Gene Ontology (GO) subgroups biological process which is shown as circles and Kyoto Encyclopedia of Genes and Genomes pathways which is shown as triangles. Terms are functionally grouped based on shared genes (kappa score) and are shown in different colours. The size of the nodes indicates the degree of significance, where the biggest nodes correspond to highest significance. The most significant term defines the name of the group. The following ClueGo parameters were used: biological process database (BP; date: 28.03.2019); GO tree levels, 2–6 (first level = 0); minimum number of genes, 3; minimum percentage of genes, 4; GO term fusion; GO term connection restriction (kappa score), 0.4; GO term grouping, initial group size of 2 and 50% for group merge. The resulting network was modified; that is, some redundant and noninformative terms were deleted and the network manually rearranged.

**Figure 5:**
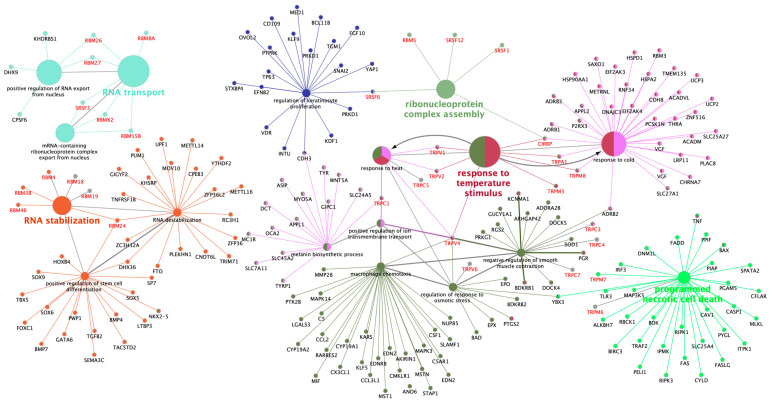
Schematic representation of functionally grouped terms with pathways and genes. Terms and their associated genes share the colour. Genes marked in red are overrepresented in all tissues and treatments. The size of circle corresponds to the *p*-value for the enrichment. This network was created using the Cytoscape v3.0.0 application and the ClueGO+CluePedia (v. 2.2.5) plug-in. The parameters included: biological process database (BP; date: 28.03.2019); Gene Ontology (GO) tree levels, 1–6 (first level = 0); minimum number of genes, 2; minimum percentage of genes, 2; GO term fusion; GO term connection restriction (kappa score), 0.4; GO term grouping, initial group size of 2 and 50% for group merge; number of genes included in term <100. The resulting network was modified to delete some redundant and noninformative terms and to manually rearrange the network.

Altered transcripts were mapped to in the Kyoto Encyclopedia of Genes and Genomes (KEGG) database [36] and were associated with GO biological process subgroups (Figure 4). Results showed that the predominant GO biological processes were response to temperature stimulus and RNA transport followed by transmembrane transport, whereas the predominant KEGG pathway was inflammatory mediator regulation of TRP channels followed by mineral absorption. Furthermore, genes were classified by functionally grouped terms and pathways (Figure 5). The enrichment *p*-values were predominant in establishment of RNA localization and response to temperature stimulus followed by response to cold, RNA stabilization, and ribonucleoprotein complex assembly.

## 3. Discussion

Relevant aspects of pig reproduction have recently focused on the differential mRNA expression evolved from the interaction between semen and the female reproductive tract [30,31,32]. However, to the best of our knowledge, the mRNA expression of genes encoding for RBPs and TRP ion channels in the sow reproductive tract is yet to be characterized, particularly whether they are induced by semen or sperm-free SP during the pre-ovulatory phase.

The RBPs are fundamental for RNA metabolism, and thereby essential to gene expression regulation [13]. A group of evolutionarily conserved RBPs, named CIPs, are transcriptionally up-regulated in response to moderately low temperatures as well as other cellular stressors [16,17]. This study explores the differential mRNA expression levels of RBPs and TRP ion channels in the reproductive tract of sows in physiological, spontaneous, stress-free estrus, following cervical deposition of semen or sperm-free SP, without any other manipulation. The study had a special interest in determining whether these cervical depositions affected the differential mRNA expression of CIPs and related TRP ion channels.

A high number of RBPs expressed in the reproductive tract regulate the expression of genes encoding for inflammatory molecules, either promoting or controlling inflammation [37]. In the present study, the majority of the DEGs encoding for RBPs were down-regulated in sperm-containing treatments compared to sperm-free SP treatments. This down-regulation could be related to an implication of RBPs on post-transcriptional regulation of immune response against spermatozoa. Thus, this signaling could induce a reduction of the response against spermatozoa retained in specific segments of the female reproductive tract considered reservoirs [38] during the pre-ovulatory phase, keeping spermatozoa safe for further reproductive events. This hypothesis is in agreement with previous research [30], which reported a down-regulation of genes related to immune pathways in the sow reproductive tract after mating. Additionally, the infundibulum showed greater numbers of up-regulated DEGs encoding for RBPs compared to proximal uterus in all treatments, perhaps related to inflammation-like changes as a pre-amble to an ovulation to be [39]. A more likely explanation, considering all the sows were in pre-ovulation oestrus stage is the fact that the porcine infundibulum depicts a particular pattern of immune reactivity in relation to sperm transport and elimination through the ostium [40].

Information is scarce about the presence of CIRBP in the reproductive tract [41] and gonads [42,43]. CIRBP can act as protein component of innate receptors in hypoxic macrophages and brain microglia interacting with MD2, the coreceptor of Toll-like receptor 4, enhancing the proinflammatory response [20,21]. Our results reported that mating up-regulated *CIRBP* mRNA expression in the uterus and the oviductal segments, suggesting that *CIRBP* may be involved in the promotion of the described inflammatory response after mating [44], but not in other treatments included in the study. Thus, our data propose that this promotion of inflammation does not result from the separate action of spermatozoa nor SP but perhaps from an intrinsic signaling mechanism maybe produced by the mating process, as it has been postulated in human as a cervico-uterine-tubal reflex [45], although never proved in pigs. As per the entry of SP, the bulk of SP does not seem to enter the oviduct of the pig, but SP proteins adsorbed to the spermatozoa do [46,47,48]. CIRBP may be involved in early reproductive processes, but more analyses are needed to clarify its role in the sow reproductive tract during the pre-ovulatory phase.

The SRSFs, which consist of 12 members, have been currently reported to regulate metabolic homeostasis and energy-dependent development [49,50]. The dysregulation of SRSFs has been related to the progression of multiple types of human tumors [51]. Here, we reported differential expression of six genes encoding for SRSFs, including *SRSF5*, a recently described mammalian CIP [16]. Our results suggest that SRSFs are involved in the interaction between the female reproductive tract and semen, particularly in the oviduct, where we found a greater number of down-regulated genes encoding for SRSFs in sperm-containing treatments. Curiously, sperm-free treatments up-regulated a greater number of genes encoding for SRSFs in the oviduct, especially in the sperm-free SP of the whole ejaculate group. Further analyses are needed to elucidate the functions of SRSFs genes in the swine female reproductive tract during the pre-ovulatory phase.

In the current study, a common down-regulation of *SRSF1* (also known as SF2/ASF), a SRSFs involved in pre-mRNA splicing [52], was reported between sperm-containing treatments in the ampulla. Even though no information is available about how *SRSF1* gene is involved in the context of porcine oocyte quality, less competent oocytes up-regulated *SRSF1* [53]. Taken together, a down-regulation of *SRSF1* might be functionally relevant to oocyte competence in the ampulla, where fertilization takes place [54].

Both SRSF3 (also called SRp20) and SRSF7 (also called 9G8) regulate 3′ untranslated region identity and length in an opposing manner [55]. Our results, mainly the ampullary down-regulation in sperm-containing treatments for *SRSF3* expression, might be related to a decrease in the arrest signaling of oocytes and embryos, as previously studied [56,57]. Moreover, SRSF3 and SRSF7 play key roles in both regulating alternative polyadenylation and splicing during mouse oocyte development [58]. Besides, SRSF7 has appeared to inhibit proliferation while enhancing apoptosis [59]. Our results regarding *SRSF7* expression were only present when sows were inseminated with the sperm-rich fraction, and solely in the oviductal tissues (utero-tubal junction, isthmus, and ampulla), thus suggesting that mating should repress, through a yet not fully understood mechanism, this apoptotic response inhibition.

The TRP ion channels are expressed in a wide variety of tissues and cells, playing fundamental roles as cellular sensors and signal integrators of an extensive Ca^2+^-mediated cellular functions maintaining ion homeostasis [60]. Investigating the differences in the number of DEGs between the treatments of the study, a greater number of up-regulated DEGs encoding for TRP ion channels were found in NM compared to the sperm-free SP of the whole ejaculate. Considering the multiple stimuli produced by mating and the resulting utero-tubal responses [2,45], it seems logical that a greater number of genes encoding for TRP ion channels were up-regulated compared to its sperm-free SP counterpart. However, at the same time, NM down-regulated a greater number of DEGs encoding for TRP ion channels compared to the rest of the treatments included in this study. This fact suggests that mating may be a complex input that is perceived by the sensory system of the female reproductive tract, including chemo-, thermo-, and mechano-sensing [27], therefore, producing a wide variety of regulatory mechanisms.

The TRPC family has seven members that can be classified in four subfamilies based on structural and functional homology: TRPC1, TRPC2, TRPC3/6/7, and TRPC4/5 [61]. All TRPC members forms Ca^2+^-permeable non-selective cation channels that display both, inward and outward rectification with reversal potentials around 0 mV [61]. The TRPC4/5 channels are activated via phospholipase C-coupled receptors, but the mechanisms of activation are still unclear [62,63]. TRPC5 is involved in the control of smooth muscle [61] and was identified as a cold sensor in neurons [64]. Previous research reported highest *TRPC5* expression in the brain compared to kidney, liver, testis, and uterus [65]. In the present study, all treatments down-regulated *TRPC5* expression in the proximal uterus. In addition, NM downregulated *TRPC5* expression in all tissue sections. Lower *TRPC5* expression has been reported in myometrium during pregnancy compared to non-pregnant rats [66]. Taking together, our results suggest that the first interaction between seminal fluid and endometrium induces a *TRPC5* down-regulation that may be involved in sperm movement through the entire female reproductive tract and perhaps could persist in myometrium during pregnancy. The involvement of TRPC5 in the control of smooth muscle and regulation of Ca^2+^ entry in myometrial cells of the female reproductive tract remains unclear [66]. However, the role of this myometrium regulation could be related to slow down the progression of the spermatozoa along the female reproductive tract after mating during the pre-ovulatory phase. In contrast, the utero-tubal junction showed a common up-regulation of *TRPC3* between NM, Semen-AI, and SP-AI.

Like TRPC5, TRPC3 is involved in the control of smooth muscle activity [61]. However, unlike TRPC5, overexpression of TRPC3 was reported in human myometrium during pregnancy and labor, compared to non-pregnant state [67]. In our case, the up-regulation of *TRPC3* could be related to the function of the sperm reservoir performed by the utero-tubal junction [1], where hyperactivated sperm motility enables the displacement of spermatozoa from the sperm reservoir to the site of fertilization during the pre-ovulatory phase.

Even though recent research identified the TRPV3, TRPV4, and TRPM8 as inductors of CIPs [16,25], no statistical differences were found for *TRPV3* and *TRPV4* expression in our study. Acting as a cold and menthol receptor, TRPM8 was identified in sensory neurons [68] and highly expressed in several tumors, particularly in prostate neoplastic cells [69]. Nevertheless, the physiological function in non-neuronal tissues is not fully understood [69]. Previous research identified the presence of TRPM8 in spermatozoa, probably involved in sperm acrosome reaction and cell signaling events, such as thermotaxis, chemotaxis, and mechanosensory transduction [70,71,72]. Here, we reported a down-regulation of *TRPM8* expression in the isthmus produced by NM. This down-regulation may be related to preserving sperm acrosome reaction in isthmus before fertilization takes place.

The TRPA1 constitutes the only mammalian member of the TRPA ion channel subfamily [73]. The TRPA1 is mainly expressed in sensory neurons as a nociceptive integrator and has been identified in multiple non-neuronal tissues [74]. However, little is known about its expression in the female reproductive tract, only being described in rat and human endometrium to date [75,76]. Non-neuronal implications of TRPA1 include inflammation, infection, and immunity [74]. Although data seems to support that TRPA1 is directly activated by cold [29], its involvement in cold sensation remains unclear [73]. Sperm-containing treatments down-regulated its expression in uterus and the lower oviductal segments (only NM), suggesting that spermatozoa may interact with TRPA1 and sensory nerves of the reproductive tract to reduce pain and neurogenic inflammation mechanically produced by mating or insemination catheter.

It seems to be certain that RBPs and TRP ion channels are involved in multiple mechanisms during the pre-ovulation phase of the sow; however, the exact mechanisms produced in the interaction between semen and the female reproductive tract have not yet been elucidated. Thus, larger, exhaustive studies are needed to clarify the detailed mechanisms of RBPs and TRP ion channels, as well as CIPs and related TRP ion channels, in the animal reproduction field.

## 4. Materials and Methods

The materials and methods followed in this study were designed according to Alvarez-Rodriguez and collaborators [30].

### 4.1. Ethics Approval

Animal handling and experimentation were performed following the European Community Directive 2010/63/EU, 22/09/2010, and current Swedish legislation (SJVFS 2017:40). The Regional Committee for Ethical Approval of Animal Experiments (Linköpings Djurförsöksetiska nämnd, Linköping, Sweden) approved the experiments. Permits number 75-12 (10/02/2012), ID1400 (02/02/2018), and Dnr 03416-2020 (26/03/2020).

### 4.2. Animals and Housing Conditions

Five young matured boars (9–11 months) of proven sperm quality (concentration, motility, and morphology) and 20 weaned sows (parity 1¨C3) of Swedish Landrace breed were obtained from a controlled breeding farm. Animals were kept in individual stalls under controlled light and temperature regimens. Animals had ad libitum access to water and were fed with commercial feedstuff.

### 4.3. Experimental Design

Segments of the female internal genital tract of 20 domestic female pigs (*Sus scrofa domestica*) were surgically retrieved under general anesthesia 24 h after treatments. mRNA expression analyses were performed on the right side of the tubular reproductive tract (uterine horns and oviducts) segmented into cervix, distal uterus, proximal uterus, utero-tubal junction, isthmus, ampulla, and infundibulum. The females were distributed (*n* = 4) to one of the following five groups:

1. Control group: females were cervically infused with protein-free BTS extender (50 mL).
2. NM: females were each mated with a single male.
3. Semen-AI: females were artificially inseminated with pooled ejaculate of the first 10 mL portion of the sperm-rich fraction (extended to 50 mL with BTS).
4. SP-TOTAL: females were cervically infused with pools of sperm-free SP of the whole ejaculate (50 mL).
5. SP-AI: females were cervically infused with the sperm-free SP harvested from pooled sperm-peak fractions (50 mL).

### 4.4. Semen and Seminal Plasma Collection

Semen was collected weekly using the gloved-hand method [77]. All ejaculates were evaluated for sperm concentration and motility. Ejaculates with at least 70% of motility and 75% of spermatozoa normal morphology were immediately used for the experiments. The SP was obtained from the whole ejaculate or the sperm-rich fraction after double centrifugation at 1500× *g* for 10 min and checking the absence of sperm cells.

### 4.5. Reproductive Management of Sows

Females were checked for pro-estrus and estrus behavioral signs twice daily using a neighboring boar as teaser and the application of back pressure by experienced staff. Sows presenting standing estrus reflex were assumed to be on the first day of behavioral estrus and were randomly assigned to control or treatment groups. Disposable conventional AI-catheters (Minitüb, Munich, Germany) were used for all cervical inseminations or infusions performed in the study [78].

### 4.6. Tissue Sample Collection

Sows were sedated and induced to general anesthesia [30] 24 h after mating, insemination, or infusion (pre-/peri-ovulation stage). The blood vessels that irrigate the female reproductive tract were clamped. The reproductive tract was opened to expose the mucosa at specific segments. Mucosal samples were retrieved and immediately plunged into liquid nitrogen (LN_2_) and stored in cryotubes at −80 °C until further analyses. The utero-tubal junction was collected as a segment and bisected, one half plunged into LN_2_ following careful cleansing with buffered saline solution and the other half fixed in 4% paraformaldehyde solution. The fixed samples were histologically processed to confirm presence of spermatozoa in animals of NM and Semen-AI groups, and alternative sperm absence in animals of SP-TOTAL, SP-AI, and Control groups. The confirmation of presence or absence of spermatozoa was done prior to use the utero-tubal junction tissues for microarrays [77,78,79]. The number of preovulatory follicles or eventual new ovulation was recorded per sow (22.30 ± 7.29, mean ± SD) without significant differences found between sow-groups.

### 4.7. Microarray Hybridization and Scanning

Total RNA from each genital tract segment was extracted following a TRIzol modified protocol [78]. Only samples with RIN values greater than 9 were used for microarray hybridization. cDNA synthesis was performed using GeneChip^®^Whole Transcript Plus reagent kit (Affymetrix, Santa Clara, CA, USA) and equal amounts of total RNA (250 ng/reaction). To make a cocktail hybridization mix for a single reaction, 3.5 μg of fragmented and labelled single-stranded cDNA (41 μL) were mixed with 109 μL of hybridization master mix. Before loading the array chip (GeneChip^®^ Porcine Gene 1.0 ST Array, Affymetrix Inc., 3420 Central Expressway, Santa Clara, CA, USA) with the hybridization cocktail, an initial incubation of the mix at 99 °C for 5 min was performed following a descent to 45 °C. Then, a total of 130 μL of the cocktail hybridization mix was loaded into each array chip and was incubated at 45 °C under 60 rotations per min, for 16 h. After the incubation, the hybridized cartridge array chip was unloaded and subjected to washing and staining using GeneChip^®^ Fluidics Station 450 (Affymetrix, Santa Clara, CA, USA), to be finally scanned using the Affymetrix GeneChip^®^ Scanner GCS3000 (Affymetrix, Santa Clara, CA, USA).

### 4.8. Microarray Data Analyses and Bioinformatics

Individual microarrays were used for each mucosal area of the female genital tract and animal. The intensity data of each array chip was treated using robust multi-array average normalization, computing average intensity values by background adjustment, quantile normalization among arrays, and finally, log_2_ transformation for extracting the expression values of each transcript in the probe set, as implemented in the Transcriptome Analysis Console (TAC, v. 4.0.) from Affymetrix (Santa Clara, CA, USA). The normalized mRNA expression data was analyzed using a linear model and the empirical Bayes’ approach implemented in the package limma. The differential expressed transcripts were calculated using a Benjamini–Hochberg false discovery rate (*q* < 0.05) and a principal component analysis-based *p*-value correction to control for multiple testing to control type-I errors.

The protein analysis through evolutionary relationships (PANTHER) GO classification system [35] was used to determine the functions of all DEGs. Pathway analysis were based on the KEGG pathway [36]. Biomolecular interaction networks were performed using the Cytoscape software [80] (v. 3.0.0.) and the ClueGo Cytoscape plug-in [81] (v. 2.0.3.).

The number of DEGs, obtained by direct comparison of all the experimental groups (NM, Semen-AI, SP-TOTAL, SP-AI) with the Control group, were analyzed by two-way ANOVA, followed by Tukey’s Multiple Comparison test using the GraphPad software for Windows v. 8.0.2. (GraphPad Software, Inc, La Jolla, CA, USA). A *p* < 0.05 was considered statistically significant.

## 5. Conclusions

In conclusion, we identified specific differentially expressed mRNA of genes encoding for CIPs and related TRP ion channels along the sow reproductive tract, suggesting that spermatozoa and seminal plasma differentially modulated both protein families during the pre-ovulatory phase. Despite absence of a clear pattern of mRNA expression regulation for the entire internal sow reproductive tract, the results suggest that these genes may play other functions than those related to cold-shock responses described in the literature, thus involved in animal reproduction probably related to an early signaling mechanism of the female reproductive tract in response to both spermatozoa and SP during the pre-ovulatory phase. Besides, the expression changes observed of other genes encoding for RBPs and TRP ion channels, such as *SRSF1*, *SRSF3*, *SRSF7*, and *TRPC3*, suggest that RBPs and TRP ion channels are involved in multiple mechanisms during the pre-ovulation phase of the sow. These results pave for larger comparative studies to clarify the detailed mechanisms of RBPs and TRP ion channels, as well as CIPs and related TRP ion channels.

## Figures and Tables

**Table 1:** Differential mRNA expression (*p* < 0.05) encoding for RNA-binding proteins and transient receptor potential ion channels in the different segments of the sow reproductive tract (cervix to infundibulum), 24 h post-treatment.

Gene Symbol	Natural Mating		Semen-AI		SP-AI		SP-TOTAL	
	UP	DOWN	UP	DOWN	UP	DOWN	UP	DOWN
*CIRBP* *	Cvx (1.62) UTJ (1.27) Amp (1.41) Inf (1.25)	**-**	-	**-**	-	**-**	-	**-**
*RBM11*		Amp (−1.91) Inf (−1.73)	-	**-**	-	**-**	-	**-**
*RBM15B*	ProxUt (1.19)	-	-	**-**	-	**-**	-	**-**
*RBM18*	-	Cvx (−1.38) Amp (−1.28) Inf (−1.26)	-	Cvx (−1.31)	-	**-**	-	Cvx (−1.22)
*RBM19*	-	-	-	-	-	DistUt (−1.15)	ProxUt (1.18) Isth (1.24)	**-**
*RBM24*	-	Isth (−1.76)	-	-	Isth (1.22)	-	-	-
*RBM25*	-	-	-	-	-	-	Inf (1.25)	-
*RBM26*	-	-	-	-	DistUt (1.11)	-	-	-
*RBM34*	-	Amp (−1.53) Inf (−1.23)	-	Inf (−1.18)	Inf (1.19)	-	UTJ (1.49)	-
*RBM38*	Cvx (1.27)	-	-	-	-	UTJ (−1.33)	-	-
*RBM39*	-	-	-	-	DistUt (1.2)	-	-	-
*RBM42*	-	-	-	Inf (−1.17)	-	Inf (−1.16)	-	-
*RBM44LOC100627002*	-	UTJ (−1.2)	UTJ (1.39)	-	-	-	-	-
*RBM47*	DistUt (1.18)			UTJ (−1.39)	-	-	-	-
*RBM5*	Cvx (1.21) UTJ (1.17) Isth (1.18) Amp (1.21) Inf (1.52)	**-**	Inf (1.21)	**-**	UTJ (1.24) Isth (1.15)	-	-	-
*RBM7*	-	DistUt (−1.4) ProxUt (−1.35) UTJ (−2) Isth (−2.48) Amp (−1.98) Inf (−2.09)	-	Cvx (−1.41), ProxUt (−1.33), Inf (−1.5)	-	-	-	-
*RBM8A*	-	Inf (−1.35)	-	Isth (−1.23) Inf (−1.25)	-	-	-	-
*RBMS1; LOC102159432*	-	DistUt (−1.23)	-	**-**	-	-	-	-
*RBMS2*	Amp (1.71) Inf (1.74)	ProxUt (−1.41)	Cvx (1.41) Isth (1.3) Amp (1.5) Inf (1.4)	**-**	-	-	-	-
*RBMX*	-	Isth (−1.18)	-	-	-	-	Inf (1.33)	-
*SRSF1*	-	Amp (−1.36)	-	Amp (−1.31)	-	-	Inf (1.43)	-
*SRSF12; LOC100739399*	DistUt (1.25)	Amp (−1.46) Inf (−1.66)	-	Isth (−1.6) Amp (−1.53) Inf (−1.68)	-	-	-	-
*SRSF3; LOC100523801; LOC106508591*	-	Amp (−1.51) Inf (−1.27)	-	UTJ (−1.26) Isth (−1.28) Amp (−1.48) Inf (−1.24)	-	--	Inf (1.5)	-
*SRSF4*	-	-	-	-	DistUt (1.14)		DistUt (1.16)	-
SRSF5; LOC100623241 *	UTJ (1.28)	-	-	Cvx (−1.21)	Inf (1.19)	-	-	-
*SRSF7*	-	-	-	UTJ (−1.25) Isth (−1.26) Amp (−1.38)	-	-	Inf (1.61)	-
TRPA1; LOC100627895 *	-	Cvx (−2.46) DistUt (−7.73) ProxUt (−6.34) Isth (−1.14)	-	DistUt (−2.47)	-	-	-	-
*TRPC1*	Inf (1.52)	ProxUt (−1.43)	-	-	-	-	-	-
*TRPC3*	UTJ (1.71)	**-**	UTJ (1.65)		UTJ (1.64)	-	-	-
*TRPC4*	ProxUt (2.23) Inf (1.29)	**-**	-	-	-	-	-	-
TRPC5 *	-	Cvx (−2.29) DistUt (−2.44) ProxUt (−3.31) UTJ (−2.65) Isth (−1.78) Amp (-1.84) Inf (−3.21)	-	ProxUt (−1.57)	-	ProxUt (−1.86)	-	ProxUt (−1.39)
*TRPC7*	Inf (1.1)	-	-	**-**	-	**-**	-	-
*TRPM3*	UTJ (1.74) Isth (3.37)	-	-	**-**	-	**-**	-	Amp (−1.68)
*TRPM6*	DistUt (2.13) ProxUt (2.2)	Inf (−1.25)	-	**-**	DistUt (1.55)	**-**	-	**-**
*TRPM7*	-	-		-	DistUt (1.24) Isth (1.12)	**-**	-	**-**
TRPM8; LOC100627434 *	-	Isth (−1.23)		-	-	**-**	-	**-**
*TRPV1*	-	**-**		Inf (−1.38)	-	**-**	-	**-**
*TRPV2*	-	**-**		Inf (−1.05)	-	**-**	-	**-**
*TRPV6*	-	**-**	Cvx (2.32)	-	-	**-**	-	**-**

Cvx: cervix; DistUt: distal uterus; ProxUt: proximal uterus; UTJ: utero-tubal junction; Isth: isthmus; Amp: ampulla; and Inf: infundibulum. UP: up-regulation and DOWN: down-regulation (in bold). The fold change for each treatment and tissue is presented between brackets. Asterisks mark genes that codify for RNA-binding proteins included in the cold-inducible proteins and genes that codify for cold-sensitive transient receptor potential ion channels. Natural mating: sows mated with a boar; Semen-AI: sows artificially inseminated with the sperm-peak portion extended to 50 mL with Beltsville thawing solution (BTS); SP-AI: sows cervically infused with the sperm-free seminal plasma (SP) from pooled sperm-peak portion (50 mL); SP-TOTAL: sows cervically infused with the sperm-free SP of the whole ejaculate (50 mL). All treatments were compared with controls.
